# Quantitative analysis of subcellular distributions with an open-source, object-based tool

**DOI:** 10.1242/bio.055228

**Published:** 2020-10-19

**Authors:** Pearl V. Ryder, Dorothy A. Lerit

**Affiliations:** Department of Cell Biology, Emory University School of Medicine, Atlanta, GA 30322, USA

**Keywords:** Centrosome, Colocalization, Image analysis, Fluorescence microscopy, RNA localization

## Abstract

The subcellular localization of objects, such as organelles, proteins, or other molecules, instructs cellular form and function. Understanding the underlying spatial relationships between objects through colocalization analysis of microscopy images is a fundamental approach used to inform biological mechanisms. We generated an automated and customizable computational tool, the SubcellularDistribution pipeline, to facilitate object-based image analysis from three-dimensional (3D) fluorescence microcopy images. To test the utility of the SubcellularDistribution pipeline, we examined the subcellular distribution of mRNA relative to centrosomes within syncytial *Drosophila* embryos. Centrosomes are microtubule-organizing centers, and RNA enrichments at centrosomes are of emerging importance. Our open-source and freely available software detected RNA distributions comparably to commercially available image analysis software. The SubcellularDistribution pipeline is designed to guide the user through the complete process of preparing image analysis data for publication, from image segmentation and data processing to visualization.

This article has an associated First Person interview with the first author of the paper.

## Abstract (precis)

**Summary:** The SubcellularDistribution pipeline is an open-source, automated, and customizable tool that guides users through image segmentation, data processing and visualization to analyze object-based colocalization within fluorescence microscopy images.

## INTRODUCTION

Microscopy imaging is a foundational technique for cell biology, as it allows for the interrogation of spatial relationships between cellular and tissue components and reveals previously unrecognized aspects of biological function (Thorn, 2016). As a general principle, the subcellular localization of biological components to specific domains is a crucial determinant of function, as spatial proximity allows for interactions among molecules, proteins, and organelles (Yang, 2013). Thus, researchers commonly investigate the localization of cellular components relative to each other using microscopy imaging to gain insights into biological function (Lagache et al., 2015). Multiple techniques detect interactions between molecules on the nanometer scale, including fluorescent resonance energy transfer (Zadran et al., 2012), fluorescence cross-correlation spectroscopy (Fitzpatrick and Lillemeier, 2011), bimolecular protein complementation (Kodama and Hu, 2012), and the proximity ligation assay (Söderberg et al., 2006). While powerful, these techniques often require specialized reagents and/or analysis and cannot detect other biologically relevant phenomena, such as indirect interactions within macromolecular complexes and direct interactions between large molecules (Lagache et al., 2015). For these reasons, investigation of the relationship between fluorescence signals remains an informative approach to infer biologically relevant interactions in fixed or live samples (Aaron et al., 2018).

An important challenge for microscopists is to extract meaningful quantitative information from data-rich images. Manual quantification of images is prone to user bias, time-intensive, may require specialized training, and may be less reproducible (Sbalzarini, 2016). Automated image analysis approaches are favored due to their reduced user bias, increased reproducibility, and ability to detect low frequency or subtle changes (Wiesmann et al., 2015).

Quantification of the relationship between fluorescence signals is referred to as colocalization analysis (Aaron et al., 2018). Colocalization analysis tests the hypothesis that two fluorescently labelled objects localize in a biological specimen within the resolution limit of the optical system (Cordelières and Bolte, 2014). In general, there are two approaches to test colocalization: methods based on the relationship between pixel intensities and methods based on the relationship between groups of pixels termed objects (Cordelières and Bolte, 2014). Pixel-based methods test for correlation between pixel intensities or spatial overlap of non-background pixels and report the degree of signal coincidence using numerical values termed colocalization coefficients (Bolte and Cordelières, 2006). However, pixel-based approaches to quantify colocalization are unable to detect signals in close physical proximity that do not overlap. Object-based approaches, on the other hand, detect the distances between objects and can reveal biologically relevant distinctions between biological conditions that may be missed by pixel-based techniques (Cordelières and Bolte, 2014).

Despite the sensitivity of object-based methods, most image analysis software platforms focus on pixel-based methods for colocalization analysis, including CellProfiler and ImageJ (McQuin et al., 2018; Schindelin et al., 2015). The JaCOP plugin for ImageJ includes object-based colocalization, but distance measurements can only be made between the centers of objects (Cordelières and Bolte, 2014). Distances between objects can be measured using CellProfiler, but the software does not provide tools to visualize the data (McQuin et al., 2018). Similarly, the Distance Transformation feature of Imaris Image Analysis Software (Imaris; Bitplane, Inc.) is a popular tool to measure distances between objects in three-dimensions (*x*, *y*, *z*; 3D), but this commercial software is cost prohibitive for some users. In addition, tools to visualize the resultant measurements are not included. Thus, the cell biology community currently lacks an open-source, customizable, and user-friendly tool to assess the distance between objects detected by fluorescence microscopy.

Here, we present the SubcellularDistribution pipeline, an automated and generalizable method to assess relationships between fluorescently labeled subcellular structures based on distance measurements between objects in 3D. This pipeline includes a single molecule normalization module that can be used to estimate the number of molecules per object for single molecule data, including single molecule RNA fluorescence *in situ* hybridization (smFISH) data. In addition, the SubcellularDistribution pipeline includes tools for data visualization. We compare our method to commercially available image analysis software and demonstrate its utility in analyzing the distribution of smFISH signals relative to two subcellular compartments, the centrosome and the nucleus. We share the SubcellularDistribution pipeline as free and open-source software with the cell biology community to facilitate robust, object-based quantification of colocalization in fluorescence microscopy images.

## RESULTS AND DISCUSSION

### Overview of the SubcellularDistribution pipeline

**Fig. 1.**
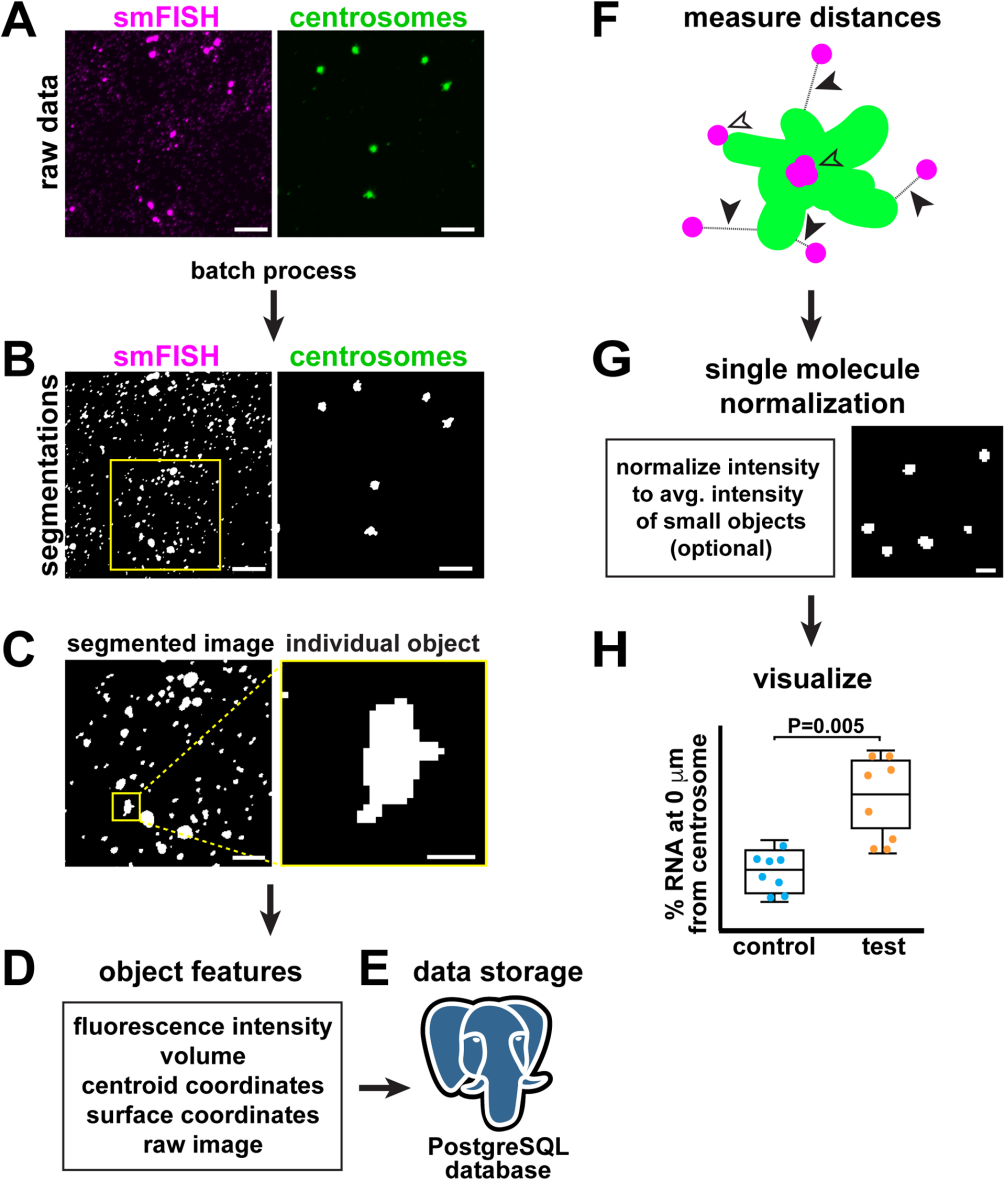
**Overview of the SubcellularDistribution pipeline.** (A) The SubcellularDistribution pipeline analyzes two or more user-provided single channel fluorescence images. Sample raw data shows *cen* mRNA (magenta; smFISH; ‘Structure 1’ in tutorial) and centrosomes labeled with GFP-Cnn (green; ‘Structure 2’ in tutorial) within a syncytial *Drosophila* embryo. (B) Images are first segmented using batch processing. Binary representations of segmented data are displayed, where foreground pixels are white and background pixels are black and excluded from further analysis. Yellow box shows region enlarged in C. (C) The segmented pixels are then grouped into individual objects. Boxed region shows inset of a single RNA object, enlarged to the right. Although segmented images of centrosomes are similarly processed, they are not shown here. (D) Next, object features are extracted. Box lists the features extracted using the SubcellularDistribution program. (E) The object feature data are stored in a PostgreSQL database. (F) Next, the distances between objects in an image are measured pairwise; open arrowheads show overlap (0 µm distance) between two objects (e.g. green centrosome versus several magenta RNA molecules), closed arrowheads show distances measured between objects. (G) An optional module is included for single molecule data to estimate the number of molecules per object using fluorescence intensity-based normalization. Image shows segmented single molecule RNA signals. (H) Finally, tools are provided to visualize data. Graph shows fictional data comparing two biological conditions (control versus test), where each dot shows the averaged measurement from a single image and a box-and-whisker plot is superimposed with midline showing the median and whiskers showing the upper and lower values. Significance was determined by a *t*-test. Scale bars: (A,B) 5 µm, (C) 2.5 µm, and (C′,G) 0.5 µm.

The SubcellularDistribution pipeline is written in Python and implemented using Jupyter notebooks and freely accessible on github (https://github.com/pearlryder/subcellular-distribution-pipeline). A detailed tutorial and a reference data set are provided to make the pipeline accessible for users who are less familiar with code-based image analysis. First, users provide single channel z-stack images of fluorescently labeled subcellular structures of interest (Fig. 1A). Analysis via the SubcellularDistribution pipeline is scalable and users can analyze as many subcellular structures as their imaging and post-processing setup can spectrally unmix. Next, images are segmented in 3D using batch processing with code adapted from the Allen Institute for Cell Science Cell Segmenter (Fig. 1B) (Chen et al., 2018 preprint). Briefly, image segmentation separates pixels into two groups: foreground pixels that are part of the structure of interest and background pixels that are not included in the analysis (Wiesmann et al., 2015). Next, groups of neighboring foreground pixels are automatically clustered into individual objects (Fig. 1C). For analysis, the SubcellularDistribution pipeline extracts the following features for each object: total pixel intensity in the original image, volume in pixels, the *x*, *y*, *z* coordinates of the centroid and the surface of the object, and the raw fluorescence image (Fig. 1D). These data are stored in a PostgreSQL relational database, which provides powerful features for parsing and analyzing the data (Fig. 1E). After object features are extracted, object coordinates are used to measure the pairwise closest distances between subcellular structures (closed arrow; Fig. 1F). Overlapping objects are assigned a distance of 0 µm (open arrows; Fig. 1F). The SubcellularDistribution pipeline includes an optional method to normalize single molecule fluorescence data (Fig. 1G). For single molecule normalization, users must predetermine the pixel volume of a single object. Finally, methods are included for visualization of the distribution of subcellular structures relative to each other (Fig. 1H). Taken together, the SubcellularDistribution pipeline offers users an open-source platform to detect, measure, and visualize distances between subcellular objects.

### The SubcellularDistribution pipeline distance measurements are comparable to commercial methods

To confirm that our image analysis pipeline was able to similarly detect the distance between subcellular structures compared to more established methods, we used our pipeline and Imaris software to analyze images of mRNA detected by smFISH and centrosomes labeled with GFP-Centrosomin in *Drosophila* embryos (GFP-Cnn; Lerit et al., 2015).

Centrosomes function as microtubule organizing centers and ensure error-free cell division and cellular organization in animal cells (Conduit et al., 2015). Centrosomes are essential for *Drosophila* embryogenesis, where thousands of centrosomes reside close to the embryonic surface amenable to a variety of imaging approaches. During the first 2 h of *Drosophila* embryo development, the syncytial nuclei undergo 13 rounds of rapid and synchronized abridged S-to-M nuclear division cycles prior to somatic cellularization at nuclear cycle (NC) 14 (Foe and Alberts, 1983). For simplicity, we will refer to M-phase embryos within metaphase as ‘metaphase’ and embryos captured pre-prophase as ‘interphase.’

**Fig. 2.**
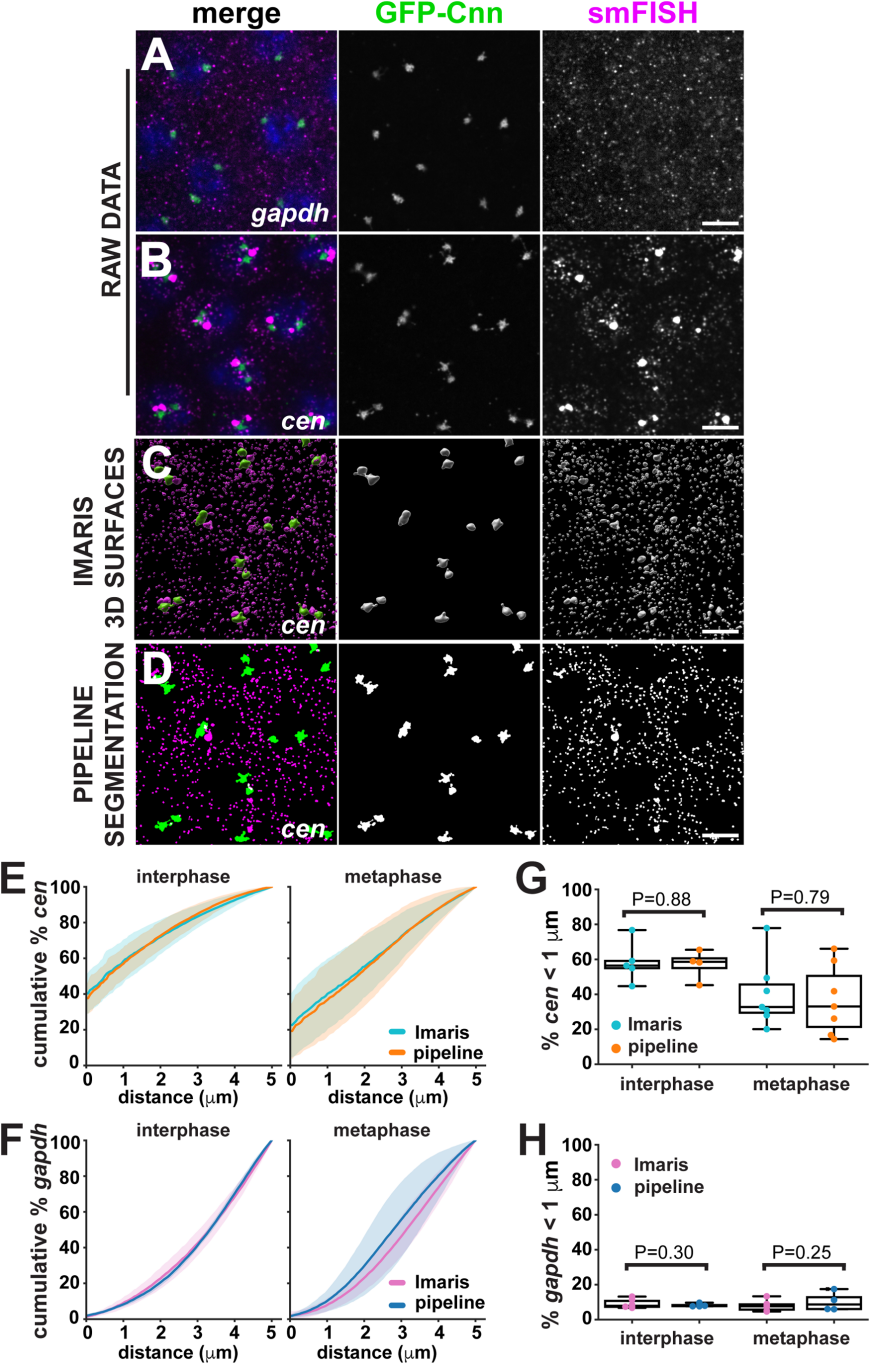
**The SubcellularDistribution pipeline is comparable to commercial software.** Images show mRNA detected by smFISH (magenta) in NC 12 embryos expressing GFP-Cnn (green). Representative images show raw data of (A) *gapdh* mRNA and (B) *cen* mRNA. In parallel analyses, raw data were segmented using (C) the Surfaces tool within Imaris software, or (D) the SubcellularDistribution pipeline. While both *gapdh* and *cen* mRNAs were processed in parallel, only *cen* is shown here. Graphs show the mean cumulative percentage of (E) *cen* mRNA and (F) *gapdh* mRNA in interphase or metaphase NC 12 embryos, where mean (dark line)±s.d. (shading) are shown relative to the distance from a centrosome. Graphs show the percent of (G) *cen* mRNA and (H) *gapdh* mRNA in interphase or metaphase NC 12 embryos localized within 1 µm of a centrosome as measured using Imaris (teal circles) or the SubcellularDistribution pipeline (orange circles), where each dot represents a measurement from one embryo and a box-and-whisker plot is superimposed. Statistical significance was tested by paired *t*-test. Scale bars: 5 µm.

Localized RNA has long been implicated as a potential regulator of the centrosome, yet remains a relatively underexplored phenomenon (Marshall and Rosenbaum, 2000; Ryder and Lerit, 2018). Work from our lab and others recently showed that *centrocortin* (*cen*) mRNA forms local enrichments near centrosomes, unlike the metabolic enzyme *gapdh* (Fig. 2A,B) (Bergalet et al., 2020; Lécuyer et al., 2007; Ryder et al., 2020 preprint).

To compare the SubcellularDistribution pipeline with Imaris, we analyzed images of *cen* and *gapdh* mRNAs from *Drosophila* NC 12 embryos. In Imaris, we segmented images of centrosomes and RNA using the Surfaces tool and measured distances using the Distance Transform tool (Fig. 2C). In parallel, we compared these reference results to measurements obtained with the SubcellularDistribution pipeline (Fig. 2D). Both methods demonstrated that *cen* mRNA is more abundant near centrosomes in interphase and metaphase embryos than *gapdh* (Fig. 2E,F). To compare results from both platforms, distance measurements were plotted by calculating the cumulative percentage of total RNA that localized up to 5 µm from a centrosome (Fig. 2E,F). We found that the mean distance (dark line) and standard deviation (lighter shading) of *cen* and *gapdh* mRNAs derived from the two methods overlapped substantially, demonstrating that these approaches generate comparable results (Fig. 2E,F). Using a paired *t*-test to statistically compare the percent of RNA residing within 1 µm of a centrosome confirmed equivalent levels of *cen* or *gapdh* RNA (no significant difference; *P*>0.05, Fig. 2G,H). We conclude that the SubcellularDistribution pipeline generates object-to-object measurements comparable to Imaris, with the advantages of being free and readily customizable. Imaris also offers a batch mode to reduce active user-time, although we found that optimal segmentation was obtained by adjusting segmentation thresholds manually for each image. In contrast, image segmentation is run in batch in the SubcellularDistribution pipeline, reducing processing time and increasing reproducibility. For reference, we processed 24 images using Imaris for the analysis presented in Fig. 2, requiring approximately 6 h of active user-time. By contrast, the same images were processed with less than 1 h of active input using the SubcellularDistribution pipeline. Actual processing times will vary depending upon the subcellular surface area and computational setup. Finally, unlike Imaris, the SubcellularDistribution pipeline includes data visualization tools, further reducing data processing time.

### Batch processing with the SubcellularDistribution pipeline using scalable datasets

Our pipeline operates via batch processing of images, which facilitates processing of large imaging datasets. We took advantage of this feature to process 81 images to investigate the distribution of another model transcript, *small ovary* (*sov*) mRNA, relative to centrosomes across multiple embryonic NCs. This analysis required less than 1 h of active user-time.

**Fig. 3.**
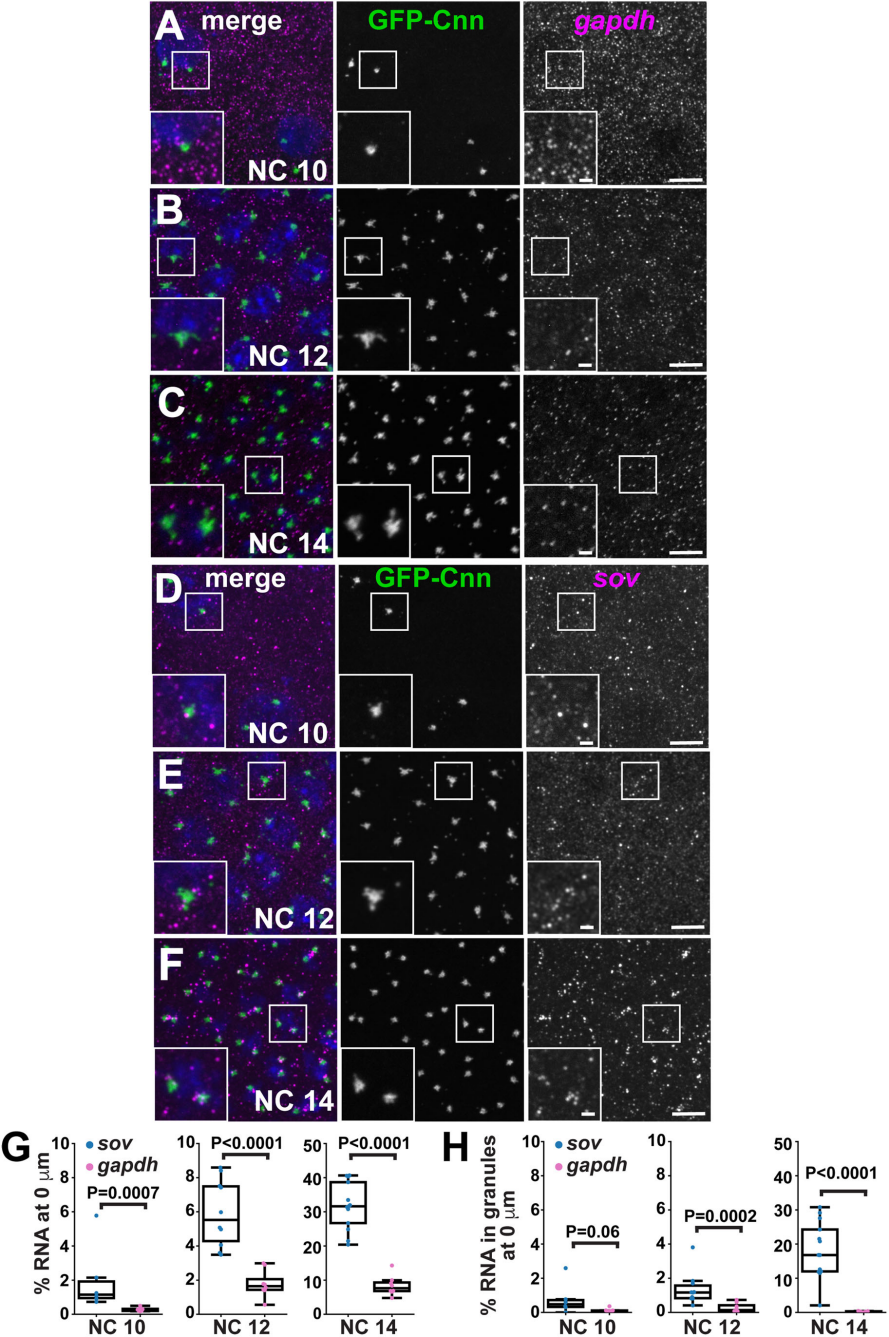
**Using the SubcellularDistribution pipeline for batch processing larger data sets.** Images show maximum intensity projections of mRNA (magenta) and centrosomes (GFP-Cnn; green) within interphase *Drosophila* embryos. The non-localizing control *gapdh* mRNA was detected in (A) NC 10, (B) NC 12, and (C) NC 14 embryos. In parallel, *sov* mRNA was detected in (D) NC 10, (E) NC 12, and (F) NC 14 embryos. (G) Graphs show the percent of RNA overlapping with centrosomes and (H) the percentage of RNA in granules overlapping with centrosomes for the indicated NC stages. Note that the axes are scaled differently for NC 14 to highlight the differences between *sov* and *gapdh*. Each dot represents a single measurement from one image, and a box-and-whiskers plot is superimposed. Significance was determined by unpaired *t*-test or the Mann–Whitney *U*-test for non-parametric data; n.s., not significant. Scale bars: 5 µm and 1 µm (insets).

Sov facilitates heterochromatin stabilization and is required for embryonic viability (Benner et al., 2019; Jankovics et al., 2018). Although Sov protein resides within the nucleus (Benner et al., 2019), *sov* mRNA localizes near centrosomes (Lécuyer et al., 2007; Ryder et al., 2020 preprint). However, the patterns of *sov* mRNA localization across different syncytial blastoderm NCs have not been closely examined. Within interphase NC 10–14 embryos, *gapdh* mRNA remained dispersed throughout the cytoplasm (Fig. 3A–C). Similarly, in NC 10 embryos, *sov* smFISH also appeared predominantly cytoplasmic (Fig. 3D). By NC 12, however, more *sov* mRNA overlapped with centrosomes (Fig. 3E). Further enrichment of *sov* was observed at centrosomes in NC 14 embryos (Fig. 3F). Analysis using the SubcellularDistribution pipeline revealed significantly more *sov* overlapping with the centrosome relative to *gapdh* in interphase NC 10–14 embryos (Fig. 3G). In NC 12 and NC 14 embryos, *sov* was also significantly enriched in higher-order RNA granules, defined as RNA objects estimated to contain greater than four individual mRNA molecules (Fig. 3H) (Little et al., 2015). Within NC 14 embryos, 28.3±11.3% of *sov* mRNA was enriched at centrosomes (*P*<0.0001; Fig. 3G), and nearly 20% of *sov* was contained within pericentrosomal granules (*P*<0.0001; Fig. 3H). These findings reveal that the localization of *sov* mRNA to centrosomes is a developmentally regulated process and may be correlated with RNA granule formation. We note peak *sov* mRNA enrichment at centrosomes during NC 14 coincides with the onset of heterochromatin formation, hinting that aspects of *sov* mRNA localization may contribute to local Sov functions (Rudolph et al., 2007; Yuan and O'Farrell, 2016). Collectively, these data demonstrate that the SubcellularDistribution pipeline facilitates investigation of the relationship between subcellular structures across scalable datasets, such as multiple developmental stages or biological conditions.

### Measuring distances between diverse subcellular structures using the SubcellularDistribution pipeline

**Fig. 4.**
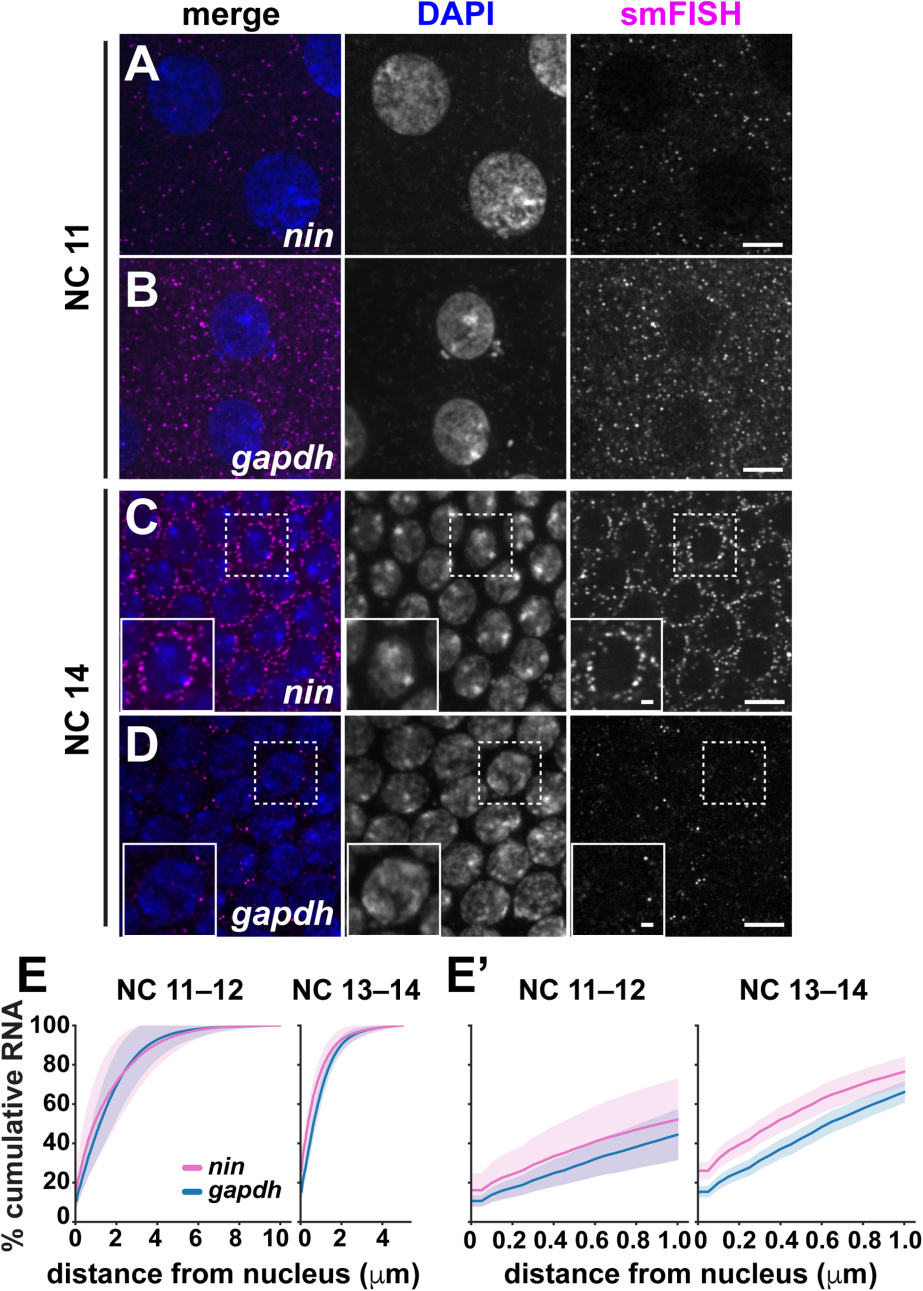
**The SubcellularDistribution pipeline detects unique subcellular localization patterns.** Images show single optical sections of mRNA (magenta) in embryos stained with DAPI to label nuclei (blue). (A) *nin* and (B) *gapdh* mRNAs appear dispersed in the cytoplasm of NC 11 embryos. Later in development, in NC 14, (C) *nin* appears concentrated in perinuclear puncta, while (D) *gapdh* remains dispersed. (E) Graphs show the cumulative distribution of RNA relative to the nuclear surface in early (NC 11 and NC 12) versus later (NC 13 and NC 14) syncytial stages from *N*=11 NC 11–12 and *N*=11 NC 13–14 embryos labeled for *gapdh* and *N*=12 NC 11–12 and *N*=12 NC 13–14 embryos labeled for *nin* mRNA. (E′) Inset of graphs highlighting differences in *gapdh* versus *nin* distribution within 1 µm of the nuclear surface. Data are plotted as mean (dark line)±s.d. (shading). Scale bars: 5 µm and 1 µm (insets).

Having established the utility of the SubcellularDistribution pipeline for measuring the distance between mRNAs and centrosomes, we next assayed its ability to detect other organelles, such as the nucleus. We examined the distribution of *Bsg25D*/*ninein* (*nin*) mRNA with smFISH. Nin is a pericentrosomal protein (Kowanda et al., 2016; Zheng et al., 2016) and *nin* mRNA localizes near centrosomes in syncytial embryos (Lécuyer et al., 2007). In NC 11 and NC 12 embryos, *nin* mRNA appeared evenly dispersed within the cytosol, similar to *gapdh* (Fig. 4A,B show representative images from NC 11 embryos; *N*=11 and *N*=12 embryos labeled for *gapdh* and *nin* mRNAs, respectively). Unexpectedly, we noted a marked enrichment of *nin* mRNA localized near nuclei in NC 13 and NC 14 embryos (Fig. 4C shows a representative image from an NC 14 embryo; *N*=12), while *gapdh* mRNA remained dispersed at this stage (Fig. 4D shows a representative image from an NC 14 embryo; *N*=11). We used the SubcellularDistribution pipeline to measure the distances between *nin* or *gapdh* mRNAs and nuclei using pooled datasets comprising images of early (NC 11 and 12) or late (NC 13 and 14) stage syncytial embryos. While NC 11–12 embryos showed no significant difference between *nin* and *gapdh* distributions, NC 13–14 embryos showed *nin* enriched near nuclei (Fig. 4E). These differences were readily detected within 1 µm of a nucleus (Fig. 4E′). We examined the relative levels of RNA residing 0 µm from a nucleus and found that 26.3±3.9% of *nin* mRNA overlaps with a nucleus, as compared to 15.5±2.6% for *gapdh* mRNA (*P*<0.0001 by unpaired *t*-test). Our data uncover an intriguing and previously unrecognized localization of *nin* mRNA within *Drosophila* embryos and reveal that *nin* mRNA redistributes to the perinuclear region during late-stage syncytial development. While the biological significance of *nin* mRNA localization to nuclei is currently unknown, Nin protein plays a key role in regulating nuclear position in *Drosophila* muscle cells (Rosen et al., 2019). Whether local *nin* mRNA resides near nuclei within muscle cells or otherwise contributes to Nin function warrants further study. Nevertheless, these findings highlight the utility of our pipeline to detect unique localization patterns between different subcellular structures. In principle, the relationship between any two or more fluorescently labelled subcellular structures may be interrogated using the SubcellularDistribution pipeline.

### Summary

The SubcellularDistribution pipeline is an open-source and freely available software designed to analyze the distribution of subcellular structures from multichannel fluorescence microscopy images. This pipeline is designed to comprehensively guide the user through the complete process of preparing data for publication, from image segmentation and data processing to visualization. As an open-source tool, users can adapt the SubcellularDistribution pipeline to their own needs. In principle, the pipeline could be used to assess the distribution of a subcellular structure relative to multiple other subcellular structures. For example, organelle–organelle contacts could be analyzed using this tool. In addition, while we have built the pipeline to focus on the distribution of fluorescence intensity between two structures, the underlying Python framework is highly adaptable. The coding savvy user could investigate other object features, such as volume or shape, relative to their distance from a subcellular structure. The pipeline also enables biologists to learn basic coding skills in Python and SQL through the reference data set and tutorial. These skills are powerful and of high value, especially for trainees approaching the job market. Ultimately, the SubcellularDistribution pipeline is a new tool to promote biological discovery by providing a framework to rigorously test the relationship between subcellular structures.

## MATERIALS AND METHODS

### Fly stocks

*P_BAC_-GFP-Cnn* flies express *cnn* tagged at the N-terminus with *GFP* under endogenous regulatory elements (Lerit et al., 2015). Flies were fed cornmeal molasses medium and maintained at 25°C in a light and temperature-controlled incubator.

### smFISH

Aged embryos (0.5–3 h) were collected on grape agar plates, and prepared for smFISH as described in Ryder et al. (2020 preprint). Briefly, embryos were fixed in a 1:4 solution of 4% paraformaldehyde:heptane for 20 min, devitellinized in methanol, and stored at −20°C or rehydrated using a stepwise series of methanol:PBST [PBS with 0.1% Tween-20), washed in wash buffer (WB: 10% formamide in 2× SSC supplemented with 0.1% Tween and 2 µg/ml nuclease-free BSA (VWR; #0332-25G)], and incubated in hybridization buffer (HB: 100 mg/ml dextran sulfate with 10% formamide in 2× SSC supplemented with 0.1% Tween, 2 µg/ml BSA, and 10 mM ribonucleoside vanadyl complex (RVC; New England Biolabs #S1402S) for 10–20 min at 37°C. Embryos were incubated overnight with Stellaris smFISH probes conjugated with either Quasar 570 or Quasar 670 dyes (Table S1) diluted 1:50 in HB, washed extensively in WB warmed to 37°C, stained with DAPI at 10 ng/ml (Thermo Fisher Scientific) in WB for 1 h, washed with PBST, and then mounted in Vectashield (Vector Laboratories; H-1000). Slides were stored at 4°C and imaged within 1 week.

### Microscopy

Images were acquired on a Nikon Ti-E system fitted with a Yokogawa CSU-X1 spinning disk head, Hamamatsu Orca Flash 4.0 v2 digital CMOS camera, Perfect Focus system, and a Nikon LU-N4 solid state laser launch (15 mW 405, 488, 561, and 647 nm) using a 100×1.49 NA Apo TIRF oil-immersion objective. This microscope was controlled through Nikon Elements AR software on a 64-bit HP Z440 workstation.

### Software installation

The use of a Docker container system to run the SubcellularDistribution pipeline facilitates the installation of the necessary software packages and avoids potential package conflicts that may arise during Python installations. Software may be accessed here: (https://hub.docker.com/r/pearlryder/subcellular-distribution-pipeline). Users are provided with detailed instructions to install Docker and download the SubcellularDistribution Pipeline: (https://github.com/pearlryder/subcellular-distribution-pipeline). A preconfigured Docker image includes the necessary software packages to run the SubcellularDistribution pipeline, including: Psycopg 2, PostgreSQL (version 11.2), Seaborn (Waskom et al., 2020), Matplotlib (Hunter, 2007), NumPy (van der Walt et al., 2011), SciPy (Virtanen et al., 2020), and pandas (McKinney, 2010). A sample data set is provided to users on FigShare to learn the SubcellularDistribution pipeline: (https://figshare.com/projects/SubcellularDistribution_pipeline/86732).

### Segmentation

Image segmentation is a binary classification of image pixels as either a component of the structure of interest or background. Our pipeline relies upon 3D-image segmentation to preserve spatial relationships captured by 3D imaging. Users may choose to segment their images using the software of their choice, including CellProfiler or Imaris (Carpenter et al., 2006). We provide instructions for segmenting images using the Allen Institute Cell Segmenter, a free and open-source software toolkit for 3D segmentation of microscopy images (Chen et al., 2018 preprint). We provide example Jupyter notebooks that are optimized for segmentation of smFISH and centrosomes. Briefly, this method prepares images for object detection by normalizing image intensities, removing background, and smoothing the images using 3D filters. Objects are identified using the Allen Institute's 3D spot filter. The watershed algorithm is applied to separate touching objects and a minimum volume threshold is applied.

### Object extraction and distance measurements

Once images are segmented, Jupyter notebooks are used to identify individual objects and extract data about each of these objects. Objects are identified in segmented images using the label method from the SciPy ndimage package (Virtanen et al., 2020). This method identifies objects as connected components where adjacent pixels share the same values. The following object features are then extracted: total pixel intensity in the original image, volume in pixels, the *x*, *y*, *z* coordinates of the object centroid and surface, and the raw fluorescence image. These features are saved in a PostgreSQL database. Distances are measured between user-specified pairs of subcellular structures, referred to here as Structure 1 and Structure 2. The pipeline first estimates distances between objects by measuring distances between the centroid coordinates of the objects. This procedure allows for a quick estimate of the closest Structure 2 object for each Structure 1 object. The distances between surface coordinates are then measured between each Structure 1 object and the three Structure 2 objects that were closest by the centroid measurements. If users are measuring distances between densely packed structures, they can increase the number of objects that are measured. This approach decreases processing time. Parallel processing is included as an option to reduce processing time.

### Single molecule RNA normalization

The SubcellularDistribution pipeline identifies single molecule objects using volume-based thresholding. For example, to normalize our smFISH data, we empirically determined that single molecules of RNA imaged with our optical system contain between 20 and 100 pixels. The pipeline calculates the average integrated fluorescence intensity of objects within these parameters, which is the average fluorescence intensity of a single molecule of RNA. To estimate the number of molecules of RNA per object, we divide each object's integrated fluorescence intensity by the average fluorescence intensity of a single molecule of RNA. This approach is adapted from Mueller et al. (2013) and Little et al. (2015).

### Visualization of RNA localization

The pipeline includes code to calculate the distribution profile of a subcellular structure relative to the distance from another subcellular structure. This calculation can be performed as fractions of Structure 1 at each distance from Structure 2 or as a cumulative distribution, where the percentage of Structure 1 that localizes within specified distances of Structure 2 is calculated for each image. For cumulative distributions, users specify a step size that determines the interval for these calculations (e.g. if users specify at step size of 0.05 μm, then the pipeline calculates the percentage of RNA localized at 0, 0.05, 0.1 μm, etc.). Users can also choose an upper distance threshold from a target object. These calculations can be saved as .csv files for plotting and/or statistical analysis. The pipeline includes code demonstrating how to plot these data using the Seaborn library (Waskom et al., 2020). Users may choose to analyze entire images or specific regions of interest, as detailed in the SubcellularDistribution pipeline github documentation.

### Statistical analysis

Statistical analysis was performed using GraphPad Prism software (version 8.4.3). To calculate significance, the distribution normality was first assessed with a D'Agnostino and Pearson normality test. Data were then analyzed by Student's two-tailed *t*-test or the appropriate nonparametric tests and are displayed as mean±s.d.

### Data management

Data tables may be exported from the Postgres database as .csv files. Instructions are also provided for saving database backup files.

## Supplementary Material

Supplementary information
