# Supplementary material for: Quantitative analysis of subcellular distributions with an open-source, object-based tool
Source: Biol Open. 2020 Oct 19;9(10):bio055228. doi: 10.1242/bio.055228 (PMC7595693; doi:10.1242/bio.055228)
Supplement: Supplementary information [file biolopen-9-055228-s1.pdf]

Table S1: List of smFISH probes

Click here to Download Table S1

Biology Open • Supplementary information
